# Development of an interpretable machine learning-based intelligent system of exercise prescription for cardio-oncology preventive care: A study protocol

**DOI:** 10.3389/fcvm.2022.1091885

**Published:** 2023-12-01

**Authors:** Tianyu Gao, Hao Ren, Shan He, Deyi Liang, Yuming Xu, Kecheng Chen, Yufan Wang, Yuxin Zhu, Heling Dong, Zhongzhi Xu, Weiming Chen, Weibin Cheng, Fengshi Jing, Xiaoyu Tao

**Affiliations:** ^1^School of Physical Education, Jinan University, Guangzhou, China; ^2^Institute for Healthcare Artificial Intelligence Application, Guangdong Second Provincial General Hospital, Guangzhou, China; ^3^Faculty of Data Science, City University of Macau, Macao, Macao SAR, China; ^4^Guangzhou Sport University, Guangzhou, China; ^5^Guangdong Women and Children Hospital, Guangzhou, China; ^6^Division of Physical Education, Guangdong University of Finance and Economics, Guangzhou, China; ^7^School of Education, City University of Macau, Macao, Macao SAR, China; ^8^School of Data Science, City University of Hong Kong, Hong Kong, Hong Kong SAR, China; ^9^Department of Industrial Engineering and Management, School of Mechanical Engineering, Shanghai Jiao Tong University, Shanghai, China; ^10^Syns Institute of Educational Research, Hong Kong, Hong Kong SAR, China; ^11^School of Public Health, Sun Yat-Sen University, Guangzhou, China; ^12^Department of Health Medicine, Guangdong Second Provincial General Hospital, Guangzhou, China; ^13^UNC Project-China, UNC Global, School of Medicine, The University of North Carolina, Chapel Hill, NC, United States; ^14^Zhuhai College of Science and Technology, Zhuhai, China; ^15^ZCST Health and Medicine Industry Research Institute, Zhuhai, China

**Keywords:** exercise prescription, machine learning, cardio-oncology, physical activity, interpretable artificial intelligence, prescription recommendation

## Abstract

**Background:**

Cardiovascular disease (CVD) and cancer are the first and second causes of death in over 130 countries across the world. They are also among the top three causes in almost 180 countries worldwide. Cardiovascular complications are often noticed in cancer patients, with nearly 20% exhibiting cardiovascular comorbidities. Physical exercise may be helpful for cancer survivors and people living with cancer (PLWC), as it prevents relapses, CVD, and cardiotoxicity. Therefore, it is beneficial to recommend exercise as part of cardio-oncology preventive care.

**Objective:**

With the progress of deep learning algorithms and the improvement of big data processing techniques, artificial intelligence (AI) has gradually become popular in the fields of medicine and healthcare. In the context of the shortage of medical resources in China, it is of great significance to adopt AI and machine learning methods for prescription recommendations. This study aims to develop an interpretable machine learning-based intelligent system of exercise prescription for cardio-oncology preventive care, and this paper presents the study protocol.

**Methods:**

This will be a retrospective machine learning modeling cohort study with interventional methods (i.e., exercise prescription). We will recruit PLWC participants at baseline (from 1 January 2025 to 31 December 2026) and follow up over several years (from 1 January 2027 to 31 December 2028). Specifically, participants will be eligible if they are (1) PLWC in Stage I or cancer survivors from Stage I; (2) aged between 18 and 55 years; (3) interested in physical exercise for rehabilitation; (4) willing to wear smart sensors/watches; (5) assessed by doctors as suitable for exercise interventions. At baseline, clinical exercise physiologist certificated by the joint training program (from 1 January 2023 to 31 December 2024) of American College of Sports Medicine and Chinese Association of Sports Medicine will recommend exercise prescription to each participant. During the follow-up, effective exercise prescription will be determined by assessing the CVD status of the participants.

**Expected outcomes:**

This study aims to develop not only an interpretable machine learning model to recommend exercise prescription but also an intelligent system of exercise prescription for precision cardio-oncology preventive care.

**Ethics:**

This study is approved by Human Experimental Ethics Inspection of Guangzhou Sport University.

**Clinical trial registration:**

http://www.chictr.org.cn, identifier ChiCTR2300077887.

## 1 Introduction

Globally, cardiovascular disease (CVD) ranks as the first leading cause of death, while cancer ranks as the second in around 130 countries (1). These two factors are also among the top three killers in almost 180 countries worldwide (2). People living with cancer (PLWC) usually exhibit cardiovascular complications resulting from so-called “cardio-toxicity” (3) (which is defined as any heart damage arising from cancer treatments) as well as the overlap of risk factors of cancer and CVD, including an unbalanced fat diet, alcohol abuse, and physical inactivity (4).

Interventions using some common prevention strategies for these risk factors exist. For example, regularly engaging in physical activity (i.e., exercise prescription) is an efficient prevention strategy for cardio-oncology (5) because physical exercise can reduce not only cardio-toxicity but also the adverse effects of chemotherapy, including lymphoedema, fatigue, and immunological disorders (6). As a result, among PLWC or cancer survivors, physical exercise is a valuable tool for CVD prevention. Therefore, it is beneficial to recommend exercise prescription in cardio-oncology preventive care (5).

Artificial Intelligence (AI) has been widely employed in healthcare and medicine, especially since great advancements in deep learning algorithms and significant improvements to big data processing techniques (7). The fields of mental health (8), internal medicine (9), infectious diseases control (10), heart failure (11, 12), and diabetes (13), among others employ AI. In the context of the shortage of medical resources in China, developing prescription recommendation systems using AI and machine learning methods is promising (14). Wang et al. have proposed a reinforcement learning-based dynamic prescription recommendation system (15).

As for a dynamic recommendation system of exercise prescription, Tuka and Linhart discussed the possibility of utilizing AI and machine learning approaches for personalized exercise prescription recommendations for patients (16). More specifically, Chen et al. presented a hierarchical learning framework for Chinese kids’ physical exercise prescription (17). However, there is no existing studies have examined machine learning-based exercise prescription recommendations for cardio-oncology preventive care. Therefore, we aim to develop an interpretable machine learning-based intelligent system of exercise prescription for cardio-oncology preventive care and present the study protocol.

## 2 Methods and analysis

### 2.1 Design

**FIGURE 1:**
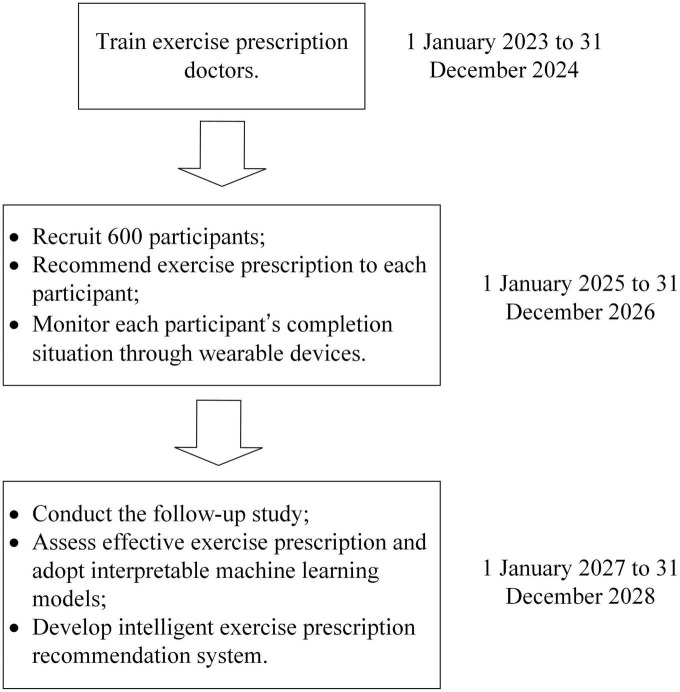
The timeline of this study.

This will be a cohort study with retrospective machine learning modeling. The study timeline is presented in Figure 1.

From 1 January 2023 to 31 December 2024, we will train our exercise prescription doctors in the training program for clinical exercise physiologist (CEP). This training program is jointly supported by American College of Sports Medicine (ACSM) and Chinese Association of Sports Medicine (CASM). Candidates who are admitted to this training program should either hold a bachelor’s degree (or above) in medicine/public health or have at least 3-year professional clinical experience in healthcare. Additionally, candidates are supposed to have a vast knowledge of physical education and sports training as well. In this training program, candidates need to complete reading materials, online courses, offline tutorials, practice, and examinations. The offline training activities will take place at Zhuhai, China, where a certificated training base of ACSM-CASM CEP programs locates on. After completion of all modules, our exercise prescription doctors will be certificated jointly by ACSM and CASM as CEP. From 1 January 2025 to 31 December 2026, we will recruit 600 participants who are PLWC (in Stage I) or cancer survivors (from Stage I) for this study. The baseline characteristics including demographics, cancer-related information, exercise habits and lifestyle, health-related physical fitness, and CVD-related items will be collected based on physical examination or biomedical testing, where necessary. Then the exercise prescription doctors certificated by ACSM-CASM will recommend exercise prescription to each participant based on the abovementioned variables. During the 2-year exercise intervention period, we will monitor each participant’s completion status through wearable devices, which can record one’s physical activity every day. Additionally, our health management team will keep track of participants every week to ensure their adherence. We will undertake the follow-up study between 1 January 2027 and 31 December 2028, re-examining CVD-related items for all participants. Finally, effective exercise prescription will be assessed based on the changes in CVD-related items from the baseline to the follow-up, and interpretable machine learning models will be adopted in effective exercise prescription. The intelligent exercise prescription recommendation system will be developed based on some machine learning models with both good interpretability and high performance.

### 2.2 Selection of subjects

This protocol involving human participants was reviewed and approved by the Ethics Committee of Guangzhou Sport University. The participants will be provided with their written informed consent to participate in this study.

#### 2.2.1 Inclusion criteria

Participants will be eligible if they are (1) PLWC in Stage I or cancer survivors from Stage I; (2) aged between 18 and 55 years; (3) interested in physical exercise for rehabilitation; (4) willing to wear smart sensors/watches; (5) assessed by doctors as suitable for exercise interventions.

#### 2.2.2 Exclusion criteria

Participants will be ineligible for inclusion if they have: (1) current or recent serious sports injuries; or (2) existing severe CVD; or (3) other conditions that may not be suitable for exercise interventions, as assessed by doctors.

### 2.3 The sample size

We will recruit 600 participants for this study. Generally, the minimum sample size for machine learning modeling is 200 (18). We estimate the loss rate in the follow-up is 47% according to a finding that 53% cancer survivors do not follow the recommended physical activity guidelines (19). We infer that 65% of exercise prescriptions will be assessed as effective for cardio-oncology preventive care after the follow-up and used for machine learning modeling. This assumed value is based on the effectiveness rate of exercise prescriptions in reducing the risk of cardiovascular events among cancer patients (20). Therefore, at least 200÷65%÷53%=580.55 participants are needed. As a result, we decide to recruit 600 participants in the baseline to ensure the guarantee the minimum sample size for machine learning modeling at last.

### 2.4 The baseline and follow-up

In the baseline and the follow-up, five aspects of characteristics/variables will be collected: demographics, cancer-related information, exercise habits and lifestyle, health-related physical fitness (21), and CVD-related items. Table 1 illustrates these variables in detail.

**TABLE 1:** Characteristics of participants in the baseline and at follow-up.

Category	Variables	Measurement/method	References
Demographics	Age	Electronic health record (EHR)	(22)
	Sex	EHR	(23)
	Marital status	Self-reported	(24)
	Education level	Self-reported	(25)
Cancer-related information	Cancer types	EHR	(26–31)
	Years living/diagnosed with cancer	EHR	
	Cancer treatment	EHR	
	Current medication (drugs used)	EHR	
Exercise habit and lifestyle	Smoking status	Self-reported	(32)
	Alcohol use	Self-reported	(33)
	Physical activity	IPAQ-L: International physical activity questionnaire (34)	(35)
	Diet	DHQ: diet history questionnaire (36)	(37)
	Sleep	PSQI: Pittsburgh sleep quality index (38)	(39)
Health-related physical fitness (21)	Body composition-anthropometry	Body mass index (BMI), body weight, height, muscle mass, fat mass, percentage of body fat, etc., by Inbody Device (40)	(41)
	Cardiorespiratory fitness (CRF)	Cooper 12 min run test (42)	(43)
	Muscle strength	Grips (44)	(45)
	Body flexibility	Sit-and-reach (46)	(47)
CVD-related items	Blood pressure	Medical test	(48)
	BPV: blood pressure variability	Medical test	(49)
	Resting heart rate	Medical test	(50)
	24-h ECG: premature beat, etc.	Medical test	(51)
	Echocardiography	Medical test	(52)
	Other relevant biochemical parameters	Medical test	(53)

Specifically, demographics, cancer-related information, exercise habits and lifestyle, and health-related physical fitness are used for exercise prescriptions, while CVD-related items are used to evaluate the effectiveness of exercise prescriptions after comparing them with the follow-up data.

### 2.5 Interventional methods

The interventional method for all participants is the exercise prescription, prescribed by our ACSM-CASM certificated exercise prescription doctors. The exercise intervention strategies are prescribed based on each participant’s baseline characteristics including demographics, cancer-related information, exercise habits and lifestyle, and health-related physical fitness.

From the professional perspective, exercise prescription doctors will determine the exercise dose considering three aspects: frequency, duration, and intensity (26). To be specific, doctors prescribe an exercise dose such as “3 times of exercise per week, 150 minutes in total, in moderate intensity” or “5 times of exercise per week, 75 minutes for each time, in high-intensity.” Frequency is the number of times of exercise per week, duration is the length of time in total, and intensity is decided to be high, moderate, or low. Furthermore, we can employ the concept of metabolic equivalent of task (MET) in exercise dose when considering frequency, duration, and intensity (54). For example, the above-mentioned two exercise doses are equivalent to each other, and both represent 7.5 MET-h/week.

During the 2-year exercise intervention period, wearable devices will be applied to monitor each participant’s completion status. In addition, our professional health management team (including certificated CEPs by ACSM-CASM programs and several assistants who hold a degree in public health, sports training, physical education, social work or psychology) will keep track of participants every week to ensure their adherence. The employment of wearable devices will be charged a deposit fee at the beginning which will be returned after the follow-up. Participants who are kept in track by our health management team during these 2 years will be given sports equipment (e.g., badminton rackets, yoga mats, foam rollers) for free every 6 months.

### 2.6 Data analysis and interpretable learning

Effective exercise prescriptions will be selected based on pre- and post-intervention data analysis of CVD-related items. For example, if the blood pressure variability decreases or at least does not increase, the exercise prescription can be considered effective. We will employ interpretable machine learning models to the learnings on exercise prescription recommendations for all effective prescriptions.

Demographics, cancer-related information, exercise habits and lifestyle, health-related physical fitness will be input variables, while frequency, duration, and intensity of exercise prescription will be output variables. We formulate the prescription learning process as a machine learning classification task. Explainable machine learning models such as logistics regression, support vector machine, decision tree, random forest, k-nearest neighbor, and naive Bayes classifiers will be utilized. Specifically, in the logistics regression machine learning model, the estimated values of coefficients and their standard deviations, *P*-values, and 95% confidence intervals, will provide us with the interpretability. The process of feature selection and kernels when using support vector machine may reveal the model interpretability (55). Figure presentation of trees and importance ranking of features for decision tree and random forest can lead to good explanations (56). In k-nearest neighbor algorithm, showing the k-nearest neighbors might also be explainable (57). As for Naive Bayes, it can be interpreted on the modular level and the conditional probability, then thus it will be very clear for us to understand how much each feature contributes toward a certain class prediction (58). Generally, these machine learning models may all have good potential for interpretability.

Some deep learning models will also be employed to evaluate the classification performance (accuracy, precision, recall, F-1 score, area under curve) in fivefold cross-validations, comparing them with the above-mentioned explainable machine learning models. Explanations of deep neural networks can be challenging (59), and hence to ensure that our deep learning models (convolutional neural networks, eXtreme gradient boosting, multilayer perceptron, deep residual network, DeepGBM) are more interpretable, an explainability tool named SHapley Additive exPlanations (SHAP), (60) will also be included. Recent advances in interpretability study for deep learning models have demonstrated the explainable potential for such models utilizing SHAP. For example, Zhao et al. proposed a novel SHAP scores computing algorithm for convolutional neural networks in classification (61). Meng et al. developed an integrated framework with better interpretability based on SHAP and eXtreme gradient boosting (62).

Since the intervention duration will be 2 years, there might be a great chance of loss-to-follow-up. Therefore, we set our sample size as 600 instead of 580, to cope with a higher loss rate or lower effectiveness rate than our initial estimations. If the final sample size for machine learning modeling still fails to reach the minimum bound (18), we will employ some few-shot learning algorithms (63) in such small size machine learning task to deal with this potential problem.

### 2.7 Intelligent system development

**FIGURE 2:**
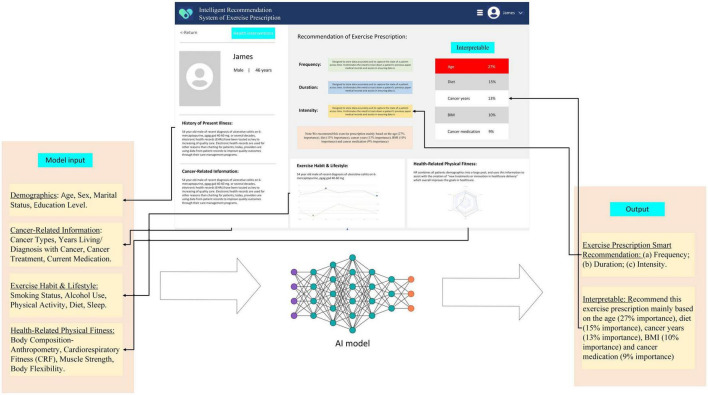
A demonstration of the intelligent system of exercise prescription.

Considering both the model performance and the model interpretability, the intelligent exercise prescription recommendation system will be developed based on some machine learning models with both good interpretability and high performance. Figure 2 depicts an example of this intelligent system. It is noteworthy that our designed intelligent system will explain why it recommends specific exercise prescriptions.

## 3 Discussion

All cancer patients need to consider a multidisciplinary approach during treatment, which includes physical training, psychological support, and lifestyle advice (26). The concept of cardio-oncology rehabilitation has been introduced by both the American Heart Association and the American Cancer Society (64). Cardio-oncology rehabilitation is to identify PLWC who are at high risk for cardiac dysfunction as well. Physical activity intervention, that is exercise prescription, is an important component of cardio-oncology rehabilitation and can prevent or moderate cardiovascular events in cancer patients or survivors. It has been demonstrated that heterogeneous responses to the same physical training can enhance cardio-respiratory fitness in cancer therapy (65). There is some theoretical and experimental evidence as to why physical exercise can help reduce cardiovascular events in cancer patients or survivors. For example, the so-called “cancer-induced cardiac cachexia,” which refers to a multi-organ/tissue syndrome affecting the brain, liver, and heart, can exist in cancer patients because of the tumor environment (66). Previous studies have revealed that physical exercise can restore muscle strength and improve endurance, thus counteracting cardiac cachexia (67). Exercise prescription can act as an aid and therapy for cardio-oncology preventive care. Therefore, our study on interpretable machine learning of exercise prescription for cardio-oncology prevention is of significance.

Some limitations of our study need to be mentioned. First, only PLWC in cancer Stage I or cancer survivors from cancer Stage I are taken into account. Although considering more participants (e.g., expansion to Stage II or Stage III) may improve our approach’s coverage, we decide to focus on participants with milder intensities of cancer. Second, there is always a trade-off between model interpretability and model performance. Black-box deep learning models may outperform explainable machine learning models in evaluation metrics such as accuracy and area under curve (AUC). Therefore, we plan to adopt both in this study and select a balanced one for intelligent system development. Third, we will only employ internal cross-validation methods in training and testing as an initial validation choice. To fill in this gap, after the interpretable machine learning-based intelligent system of exercise prescription has been developed and used in real case for a period, we will then conduct a quasi-experimental trial for external validation in another study, just like other machine learning-based medical studies did for external validation (68–70). Furthermore, with external validation and more dataset in the future, we can continuously update the intelligent system of exercise prescription through dynamic optimization of parameters of the interpretable machine learning model.

In conclusion, physical exercise is a promising interventional strategy for cancer patients or survivors during and after medical treatment and may also be effective in counteracting some adverse effects of the tumor environment or drugs on their cardiovascular system. When prescribing exercise, we need to take the cancer patients’ or survivors’ individual characteristics, cancer drugs/medications, personal lifestyle history, and health-related physical fitness into consideration. Such a tailored exercise prescription process can be learned by interpretable models using machine learning approaches and can generate an intelligent recommendation system of exercise prescription for cardio-oncology preventive care in the future.

## Ethics statement

The protocol has received approval from the Ethics Inspection of the Ethical Review Board of Guangzhou Sport University (Ethics Approval ID: 20232022-41). The patients/participants provided their written informed consent to participate in this study.

## Author contributions

TG, HR, FJ, and XT conceived the study and drafted the manuscript. FJ and XT were involved in the study design and methodology. WMC and WBC supervised the study. SH, DL, YX, KC, YW, YZ, HD, ZX, WMC, and WBC provided the insightful ideas and critically revised the manuscript. All authors contributed to the article and approved the submitted version.

## References

[1] Bray F Laversanne M Cao B Varghese C Mikkelsen B Weiderpass E Comparing cancer and cardiovascular disease trends in 20 middle-or high-income countries 2000–19: a pointer to national trajectories towards achieving Sustainable Development goal target 3.4. *Cancer Treat Rev.* (2021) 100:102290. 10.1016/j.ctrv.2021.102290 34536729 PMC8533484

[2] Bray F Laversanne M Weiderpass E Soerjomataram I. The ever-increasing importance of cancer as a leading cause of premature death worldwide. *Cancer.* (2021) 127:3029–30. 10.1002/cncr.33587 34086348

[3] Dong M Yu T Zhang Z Zhang J Wang R Tse G ICIs-related cardiotoxicity in different types of cancer. *J Cardiovasc Dev Dis.* (2022) 9:203. 10.3390/jcdd9070203 35877565 PMC9324462

[4] Koene RJ Prizment AE Blaes A Konety SH. Shared risk factors in cardiovascular disease and cancer. *Circulation.* (2016) 133:1104–14. 10.1161/CIRCULATIONAHA.115.020406 26976915 PMC4800750

[5] Wang HL Cousin L Fradley MG Donovan KA Smith B Szalacha L Exercise Interventions in cardio-oncology populations: a scoping review of the literature. *J Cardiovasc Nurs.* (2021) 36:385–404. 10.1097/JCN.0000000000000664 32195686

[6] Schmielau J Rick O Reuss-Borst M Kalusche-Bontemps EM Steimann M. Rehabilitation of cancer survivors with long-term toxicities. *Oncol Res Treat.* (2017) 40:764–71. 10.1159/000485187 29183022

[7] Rajpurkar P Chen E Banerjee O Topol EJ. AI in health and medicine. *Nat Med.* (2022) 28:31–8. 10.1038/s41591-021-01614-0 35058619

[8] Xu Z Zhang Q Li W Li M Yip PSF. Individualized prediction of depressive disorder in the elderly: a multitask deep learning approach. *Int J Med Inform.* (2019) 132:103973. 10.1016/j.ijmedinf.2019.103973 31569007

[9] Xu Z Cheng W Li Z Tse G Jing F Lian W Mass screening for low bone density using basic check-up items. *IEEE Transactions on Computational Social Systems.* Manhattan, NJ: IEEE (2022). p. 1–8. 10.1109/TCSS.2022.3176652

[10] Jing F Ye Y Zhou Y Ni Y Yan X Lu Y Identification of key influencers for secondary distribution of HIV self-testing among Chinese MSM: a machine learning approach. *Medrxiv [preprint].* (2021): 10.1101/2021.04.19.21255584 PMC10704319 37995110

[11] Tse G Zhou J Woo SWD Ko CH Lai RWC Liu T Multi-modality machine learning approach for risk stratification in heart failure with left ventricular ejection fraction ≤ 45%. *ESC Heart Fail.* (2020) 7:3716–25. 10.1002/ehf2.12929 33094925 PMC7754744

[12] Ren H Sun Y Xu C Fang M Xu Z Jing F Predicting acute onset of heart failure complicating acute coronary syndrome: an explainable machine learning approach. *Curr Probl Cardiol.* (2022) 48:101480. 10.1016/j.cpcardiol.2022.101480 36336116

[13] Zhou J Liu X Chou OH Li L Lee S Wong WT Lower risk of gout in sodium glucose cotransporter 2 (SGLT2) inhibitors versus dipeptidyl peptidase-4 (DPP4) inhibitors in type-2 diabetes. *Rheumatology (Oxford).* (2022):keac509. [Epub ahead of print]. 10.1093/rheumatology/keac509 36066415

[14] Liu C Liu X Wu F Xie M Feng Y Hu C. Using artificial intelligence (Watson for Oncology) for treatment recommendations amongst Chinese patients with lung cancer: feasibility study. *J Med Internet Res.* (2018) 20:e11087. 10.2196/11087 30257820 PMC6231834

[15] Wang L Zhang W He X Zha H. Supervised reinforcement learning with recurrent neural network for dynamic treatment recommendation. *Proceedings of the 24th ACM SIGKDD International Conference on Knowledge Discovery & Data Mining.* New York, NY: ACM (2018). p. 2447–56. 10.1145/3219819.3219961

[16] Tuka V Linhart A. Personalised exercise prescription: finding the best for our patients. *Eur J Prevent Cardiol.* (2020) 27:1366–8. 10.1177/2047487319884376 31640416

[17] Chen G Song G Wu Q. A hierarchical learning framework for Chinese kids physical exercise prescription. *Proceeding of the 2017 International Conference on Machine Learning and Cybernetics (ICMLC).* Vol. 1. Manhattan, NJ: IEEE (2017). p. 279–84. 10.1109/ICMLC.2017.8107777

[18] Cui Z Gong G. The effect of machine learning regression algorithms and sample size on individualized behavioral prediction with functional connectivity features. *Neuroimage.* (2018) 178:622–37. 10.1016/j.neuroimage.2018.06.001 29870817

[19] Blanchard CM Courneya KS Stein K. Cancer survivors’ adherence to lifestyle behavior recommendations and associations with health-related quality of life: results from the American Cancer Society’s SCS-II. *J Clin Oncol.* (2008) 26:2198–204. 10.1200/JCO.2007.14.6217 18445845

[20] Palomo A Ray RM Johnson L Paskett E Caan B Jones L Associations between exercise prior to and around the time of cancer diagnosis and subsequent cardiovascular events in women with breast cancer: a Women’s Health Initiative (WHI) analysis. *J Am Coll Cardiol.* (2017) 69:1774–1774. 10.1016/S0735-1097(17)35163-X28385306

[21] Liguori G American College of Sports Medicine [ACSM]. *ACSM’s Guidelines for Exercise Testing and Prescription.* Philadelphia, PA: Lippincott Williams & Wilkins (2020).

[22] Ingram DK. Age-related decline in physical activity: generalization to nonhumans. *Med Sci Sports Exerc.* (2000) 32:1623–9. 10.1097/00005768-200009000-00016 10994915

[23] Parker BA Kalasky MJ Proctor DN. Evidence for sex differences in cardiovascular aging and adaptive responses to physical activity. *Eur J Appl Physiol.* (2010) 110:235–46. 10.1007/s00421-010-1506-7 20480371 PMC2929283

[24] Pettee KK Brach JS Kriska AM Boudreau R Richardson CR Colbert LH Influence of marital status on physical activity levels among older adults. *Med Sci Sports Exerc.* (2006) 38:541–6. 10.1249/01.mss.0000191346.95244.f7 16540843

[25] Shaw BA Spokane LS. Examining the association between education level and physical activity changes during early old age. *J Aging Health.* (2008) 20:767–87. 10.1177/0898264308321081 18559963 PMC2570711

[26] D’Ascenzi F Anselmi F Fiorentini C Mannucci R Bonifazi M Mondillo S. The benefits of exercise in cancer patients and the criteria for exercise prescription in cardio-oncology. *Eur J Prevent Cardiol.* (2021) 28:725–35. 10.1177/2047487319874900 31587570

[27] Luan X Tian X Zhang H Huang R Li N Chen P Exercise as a prescription for patients with various diseases. *J Sport Health Sci.* (2019) 8:422–41. 10.1016/j.jshs.2019.04.002 31534817 PMC6742679

[28] Codima A das Neves Silva W de Souza Borges AP de Castro G. Exercise prescription for symptoms and quality of life improvements in lung cancer patients: a systematic review. *Support Care Cancer.* (2021) 29:445–57. 10.1007/s00520-020-05499-6 32388616

[29] Kirkham AA Bonsignore A Bland KA McKenzie DC Gelmon KA Van Patten CL Exercise prescription and adherence for breast cancer: one size does not FITT all. *Med Sci Sports Exerc.* (2018) 50:177–86. 10.1249/MSS.0000000000001446 28991038

[30] Hayes SC Spence RR Galvão DA Newton RU. Australian association for exercise and sport science position stand: optimising cancer outcomes through exercise. *J Sci Med Sport.* (2009) 12:428–34. 10.1016/j.jsams.2009.03.002 19428291

[31] Sweegers MG Altenburg TM Chinapaw MJ Kalter J Verdonck-de Leeuw IM Courneya KS Which exercise prescriptions improve quality of life and physical function in patients with cancer during and following treatment? A systematic review and meta-analysis of randomised controlled trials. *Br J Sports Med.* (2018) 52:505–13. 10.1136/bjsports-2017-097891 28954800

[32] Kaczynski AT Manske SR Mannell RC Grewal K. Smoking and physical activity: a systematic review. *Am J Health Behav.* (2008) 32:93–110. 10.5993/AJHB.32.1.9 18021037

[33] Dodge T Clarke P Dwan R. The relationship between physical activity and alcohol use among adults in the United States: a systematic review of the literature. *Am J Health Promot.* (2017) 31:97–108. 10.1177/0890117116664710 27630108

[34] Van Holle V De Bourdeaudhuij I Deforche B Van Cauwenberg J Van Dyck D. Assessment of physical activity in older Belgian adults: validity and reliability of an adapted interview version of the long International Physical Activity Questionnaire (IPAQ-L). *BMC Public Health.* (2015) 15:433. 10.1186/s12889-015-1785-3 25928561 PMC4427934

[35] Teixeira PJ Carraça EV Markland D Silva MN Ryan RM. Exercise, physical activity, and self-determination theory: a systematic review. *Int J Behav Nutr Phys Act.* (2012) 9:1–30. 10.1186/1479-5868-9-78 22726453 PMC3441783

[36] Millen AE Midthune D Thompson FE Kipnis V Subar AF. The National Cancer Institute diet history questionnaire: validation of pyramid food servings. *Am J Epidemiol.* (2006) 163:279–88. 10.1093/aje/kwj031 16339051

[37] World Health Organization [WHO]. *Global Strategy on Diet, Physical Activity and Health.* Geneva: World Health Organization (2004).

[38] Buysse DJ Reynolds CF III Monk TH Berman SR Kupfer DJ. The Pittsburgh sleep quality index: a new instrument for psychiatric practice and research. *Psychiatry Res.* (1989) 28:193–213. 10.1016/0165-1781(89)90047-4 2748771

[39] Semplonius T Willoughby T. Long-term links between physical activity and sleep quality. *Med Sci Sports Exerc.* (2018) 50:2418–24. 10.1249/MSS.0000000000001706 30048409

[40] Antonio J Kenyon M Ellerbroek A Carson C Burgess V Tyler-Palmer D Comparison of Dual-Energy X-Ray Absorptiometry (DXA) versus a Multi-frequency Bioelectrical Impedance (InBody 770) device for body composition assessment after a 4-week hypoenergetic diet. *J Funct Morphol Kinesiol.* (2019) 4:23. 10.3390/jfmk4020023 33467338 PMC7739224

[41] Brodie D Moscrip V Hutcheon R. Body composition measurement: a review of hydrodensitometry, anthropometry, and impedance methods. *Nutrition.* (1998) 14:296–310. 10.1016/S0899-9007(97)00474-7 9583375

[42] Bandyopadhyay A. Validity of Cooper’s 12-minute run test for estimation of maximum oxygen uptake in male university students. *Biol Sport.* (2015) 32:59–63. 10.5604/20831862.1127283 25729151 PMC4314605

[43] Grant S Corbett K Amjad AM Wilson J Aitchison T. A comparison of methods of predicting maximum oxygen uptake. *Br J Sports Med.* (1995) 29:147–52. 10.1136/bjsm.29.3.147 8800845 PMC1332303

[44] Roberts HC Denison HJ Martin HJ Patel HP Syddall H Cooper C A review of the measurement of grip strength in clinical and epidemiological studies: towards a standardised approach. *Age Ageing.* (2011) 40:423–9. 10.1093/ageing/afr051 21624928

[45] Jaric S. Muscle strength testing. *Sports Med.* (2002) 32:615–31. 10.2165/00007256-200232100-00002 12141882

[46] Baltaci G Un N Tunay V Besler A Gerçeker S. Comparison of three different sit and reach tests for measurement of hamstring flexibility in female university students. *Br J Sports Med.* (2003) 37:59–61. 10.1136/bjsm.37.1.59 12547745 PMC1724584

[47] Horsfall I Champion SM Watson CH. The development of a quantitative flexibility test for body armour and comparison with wearer trials. *Appl Ergon.* (2005) 36:283–92. 10.1016/j.apergo.2005.01.005 15854571

[48] Arroll B Beaglehole R. Does physical activity lower blood pressure: a critical review of the clinical trials. *J Clin Epidemiol.* (1992) 45:439–47. 10.1016/0895-4356(92)90093-3 1588350

[49] Xu X Meng X Oka SI. Long-term habitual vigorous physical activity is associated with lower visit-to-visit systolic blood pressure variability: insights from the SPRINT trial. *Am J Hypertens.* (2021) 34:463–6. 10.1093/ajh/hpaa198 33245323

[50] Jensen MT Suadicani P Hein HO Gyntelberg F. Elevated resting heart rate, physical fitness and all-cause mortality: a 16-year follow-up in the Copenhagen Male Study. *Heart.* (2013) 99:882–7. 10.1136/heartjnl-2012-303375 23595657 PMC3664385

[51] Hein H Panhuyzen-Goedkoop N Corrado D Hoffmann E Biffi A Delise P Recommendations for participation in leisure-time physical activity and competitive sports in patients with arrhythmias and potentially arrhythmogenic conditions Part I: supraventricular arrhythmias and pacemakers. *Eur J Prevent Cardiol.* (2006) 13:475–84. 10.1097/01.hjr.0000216543.54066.72 16874135

[52] Upshaw JN Hubbard RA Hu J Brown JC Smith AM Demissei B Physical activity during and after breast cancer therapy and associations of baseline physical activity with changes in cardiac function by echocardiography. *Cancer Med.* (2020) 9:6122–31. 10.1002/cam4.3277 32645252 PMC7476829

[53] Hosseini SRA Hejazi K. The effects of Ramadan fasting and physical activity on blood hematological-biochemical parameters. *Iranian J Basic Med Sci.* (2013) 16:845. 23997915 PMC3758056

[54] Mendes MDA Da Silva I Ramires V Reichert F Martins R Ferreira R Metabolic equivalent of task (METs) thresholds as an indicator of physical activity intensity. *PLoS One.* (2018) 13:e0200701. 10.1371/journal.pone.0200701 30024953 PMC6053180

[55] Navia-Vázquez A Parrado-Hernández E. Support vector machine interpretation. *Neurocomputing.* (2006) 69:1754–9. 10.1016/j.neucom.2005.12.118

[56] Marchese Robinson RL Palczewska A Palczewski J Kidley N. Comparison of the predictive performance and interpretability of random forest and linear models on benchmark data sets. *J Chem Inform Model.* (2017) 57:1773–92. 10.1021/acs.jcim.6b00753 28715209

[57] Rajani NF Krause B Yin W Niu T Socher R Xiong C. Explaining and improving model behavior with k nearest neighbor representations. *arXiv [preprint].* (2020):

[58] Ridgeway G Madigan D Richardson T O’Kane J. Interpretable boosted naïve bayes classification. *KDD-98 Proceedings.* New York, NY: ACM (1998). p. 101–4.

[59] Zhang Q Wu YN Zhu SC. Interpretable convolutional neural networks. *Proceedings of the IEEE Conference on Computer Vision and Pattern Recognition (8827-8836).* Manhattan, NJ: IEEE (2018). 10.1109/CVPR.2018.00920

[60] Van den Broeck G Lykov A Schleich M Suciu D. On the tractability of SHAP explanations. *J Artificial Intell Res.* (2022) 74:851–86. 10.1613/jair.1.13283

[61] Zhao W Joshi T Nair VN Sudjianto A. Shap values for explaining CNN-based text classification models. *arXiv [Preprint].* (2020):

[62] Meng Y Yang N Qian Z Zhang G. What makes an online review more helpful: an interpretation framework using XGBoost and SHAP values. *J Theor Appl Electron Comm Res.* (2020) 16:466–90. 10.3390/jtaer16030029

[63] Wang Y Yao Q Kwok JT Ni LM. Generalizing from a few examples: a survey on few-shot learning. *ACM Comput Surveys (CSUR).* (2020) 53:1–34. 10.1145/3386252

[64] Gilchrist SC Barac A Ades PA Alfano CM Franklin BA Jones LW Cardio-oncology rehabilitation to manage cardiovascular outcomes in cancer patients and survivors: a scientific statement from the American Heart Association. *Circulation.* (2019) 139:e997–1012. 10.1161/CIR.0000000000000679 30955352 PMC7603804

[65] Scott JM Nilsen TS Gupta D Jones LW. Exercise therapy and cardiovascular toxicity in cancer. *Circulation.* (2018) 137:1176–91. 10.1161/CIRCULATIONAHA.117.024671 29530893 PMC6028047

[66] Argilés JM Busquets S López-Soriano FJ Costelli P Penna F. Are there any benefits of exercise training in cancer cachexia? *J Cachexia Sarcopenia Muscle.* (2012) 3:73–6. 10.1007/s13539-012-0067-5 22565649 PMC3374018

[67] Antunes JMM Ferreira RM Moreira-Gonçalves D. Exercise training as therapy for cancer-induced cardiac cachexia. *Trends Mol Med.* (2018) 24:709–27. 10.1016/j.molmed.2018.06.002 29980479

[68] Churpek MM Carey KA Edelson DP Singh T Astor BC Gilbert ER Internal and external validation of a machine learning risk score for acute kidney injury. *JAMA Netw Open.* (2020) 3:e2012892. 10.1001/jamanetworkopen.2020.12892 32780123 PMC7420241

[69] Lu Y Ni Y Wang Q Jing F Zhou Y He X Effectiveness of sexual health influencers identified by an ensemble machine learning model in promoting secondary distribution of HIV self-testing among men who have sex with men in China: study protocol for a quasi-experimental trial. *BMC Public Health.* (2021) 21:1772. 10.1186/s12889-021-11817-2 34583667 PMC8480079

[70] Plante TB Blau AM Berg AN Weinberg AS Jun IC Tapson VF Development and external validation of a machine learning tool to rule out COVID-19 among adults in the emergency department using routine blood tests: a large, multicenter, real-world study. *J Med Internet Res.* (2020) 22:e24048. 10.2196/24048 33226957 PMC7713695

